# Endometrial Cancer and Hypermethylation: Regulation of DNA and MicroRNA by Epigenetics

**DOI:** 10.1155/2012/738274

**Published:** 2012-04-03

**Authors:** Kouji Banno, Iori Kisu, Megumi Yanokura, Kenta Masuda, Yusuke Kobayashi, Arisa Ueki, Kosuke Tsuji, Wataru Yamagami, Hiroyuki Nomura, Nobuyuki Susumu, Daisuke Aoki

**Affiliations:** Department of Obstetrics and Gynecology, Keio University School of Medicine, Shinanomachi 35 Shinjuku-ku, Tokyo 160-8582, Japan

## Abstract

Endometrial cancer is the seventh most common cancer in women worldwide. Therefore elucidation of the pathogenesis and development of effective treatment for endometrial cancer are important. However, several aspects of the mechanism of carcinogenesis in the endometrium remain unclear. Associations with genetic variation and mutations of cancer-related genes have been shown, but these do not provide a complete explanation. Therefore, in recent years, epigenetic mechanisms that do not involve changes in DNA sequences have been examined. Studies aimed at detection of aberrant DNA hypermethylation in cancer cells present in microscopic amounts *in vivo* and application of the results to cancer diagnosis have also started. Breakdown of the DNA mismatch repair mechanism is thought to play a large role in the development of endometrial cancer, with changes in the expression of the *hMLH1* gene being particularly important. Silencing of genes such as *APC* and *CHFR*, *Sprouty 2*, *RASSF1A*, *GPR54*, *CDH1*, and *RSK4* by DNA hypermethylation, onset of Lynch syndrome due to hereditary epimutation of *hMLH1* and *hMSH2* mismatch repair genes, and regulation of gene expression by microRNAs may also underlie the carcinogenic mechanisms of endometrial cancer. Further understanding of these issues may permit development of new therapies.

## 1. Introduction

Endometrial cancer is the seventh most common cancer in women worldwide. In Japan, westernization of lifestyle has increased the number of patients with endometrial cancer, and this disease now accounts for about 40% of cancers of the uterus. A further increase, and a younger onset age are also predicted, and therefore elucidation of the pathogenesis and development of effective treatment are needed. However, the mechanism of carcinogenesis in the endometrium remains unclear. Genetic aberrances such as variations in gene expression and mutation of cancer-related genes have been identified, but these do not fully explain canceration in the endometrium. Therefore, epigenetic changes in gene expression through effects on chromatin without DNA mutation are drawing attention. Breakdown of the DNA mismatch repair mechanism by aberrant DNA hypermethylation is particularly important for development of type 1 endometrial cancer, and changes in expression of genes such as human MutL homolog1 (*hMLH1*) and human MutS homolog2 (*hMSH2*) may be involved in this mechanism. The possible epigenetic mechanisms include epimutation, hypermethylation causing epimutation, and regulation of gene expression by small noncoding RNAs, called microRNAs, that bind upstream of the gene and do not change the methylation level of the gene itself. In this review, DNA hypermethylation associated with endometrial cancer based on epimutation of three genes, *hMLH1*, *hMSH2* and epithelial cell adhesion molecule (*EPCAM*), and the effects of miRNAs in endometrial cancer will be discussed.

## 2. New Findings on DNA Hypermethylation in Endometrial Cancer

Among the epigenetic mechanisms, DNA methylation has been most widely studied. Changes in DNA methylation have been associated with various tumorous lesions [1, 2], and hypermethylation is commonly associated with downregulation of gene expression. Our review in 2009 [3] prompted further studies of aberrant DNA hypermethylation associated with endometrial cancer, and it has been found that hypermethylation of gene promoters is linked to reduced expression of several genes in this cancer. We have reported silencing due to hypermethylation for *hMLH1 *and adenomatous polyposis coli (*APC*), *E-cadherin*, checkpoint with FHA and RING (*CHFR*), caspase-8 (*CASP8*), transforming growth factor, beta receptor II (*TGF-bRII*), *p73*, homeobox A11 (*HOXA11*), and catechol-O-methyltransferase (*COMT*) [3], and genes such as *Sprouty 2 *[4], Ras association domain family 1 isoform A (*RASSF1A*) [5], G-protein coupled receptor 54 (*GPR54*) [6], cadherin 1 (*CDH1*) [7], and ribosomal S6 kinase4 (*RSK4*) [8] have also been found to be silenced by a similar mechanism. Hypermethylation of the *APC* promoter is not found in the normal endometrium or in endometrial hyperplasia but is detected in atypical hyperplasia and early endometrial cancer. Interestingly, the frequency of hypermethylation in the* APC* promoter is reduced with progression of endometrial cancer, which led Ignatov et al. to suggest that this hypermethylation may be an important event in early canceration of the endometrium [9]. Satoh et al. linked hypermethylation to the response of tumors to taxane drugs [10], and Wang et al. found that reduced expression of *CHFR* by hypermethylation improves the response of both stomach and endometrial cancers to paclitaxel [11]. These studies suggest the possibility of personalized cancer treatment adapted to each patient following examination of the expression levels of multiple genes.

*Sprouty 2* (*SPRY2*) is an antagonist regulator of receptor tyrosine kinases in the fibroblast growth factor (FGF) and RAS-MAPK pathways and a tumor suppressor gene involved in cell proliferation, differentiation, and angiogenesis. Parts of the FGF and RAS-MAPK pathways are changed in endometrial cancer, and expression of *SPRY2 *is reduced due to hypermethylation in several types of cancer [12–14]. Velasco et al. found that *SPRY2* in the normal endometrium is expressed in accord with the menstrual cycle and suggested that *SPRY2 *contributes to growth of glandular structures [4]. *SPRY2* expression is extremely low in advanced invasive cancers and other types of endometrial cancer, other than endometrioid adenocarcinoma, which indicates that *SPRY2* may play a role in suppression of endometrial cancer by regulating the MAPK pathway [4].

*RASSF1A* is a tumor suppressor that is a negative regulator in the RAS-MAPK pathway and, along with *SPRY2*, has reduced expression in various cancers. In a comparison of normal and cancerous endometrial tissues, Pallares et al. showed that *RASSF1A* promoter hypermethylation and reduced expression were particularly prevalent in endometrial cancer with microsatellite instability, especially in advanced cancers [5]. This led to the suggestion that *RASSF1A* participates in cell proliferation and apoptosis by regulating the MAPK pathway and has effects on canceration of the endometrium [5].

*GPR54* is a gene-encoding endogenous receptor of *kisspeptin* (*KISS1*), a suppressor of cancer metastasis, and has reduced expression in some cancers, but an unclear status in endometrial cancer. Kang et al. found high survival rates in cases with high *GPR54 *expression and showed that expression of *GPR54 *is epigenetically regulated [6]. Functional recovery of *GPR54 *was possible with treatment with 5-aza-dC, a drug that causes DNA hypomethylation, and subsequent expression of *GPR54* and activation of a downstream response pathway involving metastin-10 were effective for inhibiting metastasis of endometrial cancer [6].

*CDH1* is a promoter of *E-cadherin*. Yi et al. found that hypermethylation of *CDH1 *reduced *E-cadherin* expression in endometrial cancer, with resulting effects on clinical and pathological progression and 5-year survival rates. Hypermethylation of *CDH1 *is common in undifferentiated cancers, leading to the suggestion that this mechanism may be closely associated with canceration and invasive capacity [7].

*X-linked Ribosomal S6 Kinase RPS6KA6 *(*RSK4*) is a substrate of ERK that inhibits transcriptional activity of some receptor tyrosine kinases for certain targets. *RSK4* is also associated with the *FGFR2/RAS/ERK* signal pathway and is a tumor suppressor. Dewdney et al. showed that expression of *RSK4* is reduced by hypermethylation in colon, breast, and kidney cancer, as well as in endometrial cancer, but the tumor inhibitory action of *RSK4* in the endometrium is unclear [8].

## 3. Epimutation and Carcinogenesis of the Endometrium

Epimutation refers to the epigenetic silencing of a gene for which expression is normally not suppressed, or epigenetic activation of a gene for which expression is normally suppressed [15, 16]. Studies of canceration of the endometrium and epimutation of genes have mainly focused on *hMLH1*, *hMSH2*, and *EPCAM*. *hMLH1* and *hMSH2* are DNA mismatch repair (MMR) genes that have a strong association with endometrial cancer, above that of other MMR genes such as *hMSH6* and *PMS2* [17]. Kondo et al. first showed that epigenetic inhibition of *hMLH1* expression is more frequent than that of *hMSH2 *in endometrial cancer [18]. Most subsequent studies have examined *hMLH1,* and this gene has been found to be a tumor suppressor that has reduced expression in various cancers. Mutation of *hMLH1* is especially common in cases with multiple primary cancers such as, for example, in Lynch syndrome (hereditary nonpolyposis colorectal cancer: HNPCC), and epimutation of the germline may be present even if mutation of *hMLH1* itself does not occur [19]. Hitchins et al. suggested that the epimutation may be inherited through the mother since during egg formation an epigenetic error can essentially be solved by demethylation; however, this type of heredity epimutation occurs with lower frequency than the germline mutation [20]. However, in one of two cases in which epimutation of *hMLH1* occurred* de novo*, Goel et al. showed that the epimutation occurred in the allele derived from the father, rather than the mother [21]. This defect may have occurred during egg formation without demethylation, and the epimutation may have been inherited or may have occurred as a new epimutation after fertilization.

An inherited germline epimutation of* hMSH2* was reported in 2006 by Chan et al. [22] in a family line of three patients (brothers and sisters) with colon or endometrial cancer with onset at an early age. Genetic mutation of *hMSH2* was not present, but protein deficiency was recognized, and microsatellite instability was shown, with the epimutation of *hMSH2* inherited from the mother. The same epimutation was inherited by three children of the three patients, indicating that not only a DNA sequence mutation but also an epimutation can be inherited over multiple generations. Sequencing shows that the epimutation does not occur in all cells and methylation mosaics exist. Different degrees of methylation occur based on the two-hit theory and can be the cause of heritability of diseases.

The *EPCAM* gene codes for an epithelial cell adhesion molecule and is overexpressed in most cancers. There is a diversity of opinion on the effects of *EPCAM* on canceration. EPCAM is a homophilic intercellular adhesion molecule that may prevent metastasis, but conversely EPCAM may also promote metastasis of cancers to prevent intercellular adhesion mediated by E-cadherin [23]. In endometrial cancer, EPCAM deficiency is also involved in hypermethylation of the *hMSH2* promoter [24]. Ligtenberg et al. found that epimutation of *EPCAM* itself is not involved in development of endometrial cancer, but a mutation to the 3′ side of* EPCAM *upstream and close to *hMSH2* epigenetically silences *hMSH2* and may lead to endometrial cancer and Lynch syndrome.

## 4. Endometrial Cancer and Regulation with Aberrant Methylation of MicroRNA

MicroRNAs (miRNAs) are short noncoding RNAs of about 18–25 bases that regulate expression of genes. miRNAs have been found to be downregulated by methylation of DNA in various cancers, and these miRNAs are referred to as tumor suppressor miRNAs (TS-miRNAs) [25, 26]. Identified TS-miRNAs include *miR-124*, *miR-126*, *miR-137*, and *miR-491* [25–29]. Using a microarray assay, Huang et al. found that *SRY-related high-mobility group box 4 (SOX4)* genes were highly expressed in endometrial cancer cells. In database analysis, *SOX4* was identified as a target for *miR-129-2*, and a reporter assay showed that *miR-129-2* was a negative regulator of *SOX4*. Expression of *miR-129-2 *was verified in 117 cases of endometrial cancer with increased expression of *SOX4*, and in 68% of the patients *SOX4 *expression was silenced by methylation. Histone acetylation and DNA demethylation were found to increase the expression of *miR-129-2*, reduce *SOX4 *expression, and suppress proliferation of cancer cells. Furthermore, hypermethylation of the *miR-129-2* gene was statistically related to microsatellite instability and the methylation status of *hMLH1* [30].

Expression of *miR-152* is also inhibited by DNA hypermethylation [31]. Tsuruta et al. identified *miR-152 *as a candidate TS-miRNA in endometrial cancer through DNA methylation screening and expression screening of endometrial cancer cells. Methylation of *miR-152* is altered in acute lymphocytic leukemia, and *miR-152* expression is changed in digestive system cancers and cholangiocellular cancer [32–34]. Tsuruta et al. found methylation and downregulation of *miR-152* at high frequency and showed that expression of *miR-152 *is recovered by 5-aza-dC. Methylation of *miR-152* is completely consistent with expression, and hypermethylation in the promoter region of *miR-152* reduces the expression. *DNMT1* is a well-known target of *miR-152*, and *E2F3*, *MET,* and *Rictor* have been identified as additional targets [34].* E2F3* is an E2F family transcriptional inhibitor and may be a cancer gene [35].* MET* is a gene that encodes a cell-surface receptor for hepatocyte growth factor and a known cancer gene [36].* Rictor* is a component of *mTORC2* (*mTOR complex 2*) which directly regulates phosphorylation of *Akt* and is important in cancer cells [37, 38]. Activation of *mTORC2-Akt *signaling contributes to canceration of the endometrium, but the action of *Rictor* in canceration is unclear. However, silencing of *miR-152* appears to lead to activation of multiple targets, and *miR-152* itself and *E2F3*, *MET,* and *Rictor* may be new targets for treatment of endometrial cancer [31].

## 5. Conclusion

In 2007, endometrial cancer was newly diagnosed in as many as 226,000 women worldwide and is the cause of death of more than 7000 people annually in the USA alone. In Japan, the number of patients with this cancer is increasing and a younger age of onset is also predicted, which emphasizes the need to find more effective therapies based on improved understanding of the pathogenesis. As for many other cancers, development of endometrial cancer cannot be completely explained by genetic mutations alone and is likely to involve epigenetic changes. In endometrial cancer, mutations of MMR genes are important, and regulation of these genes has been widely examined. Expression of *hMLH1*, *hMSH2*, and *EPCAM* is suppressed by promoter hypermethylation, which inhibits direct or indirect DNA mismatch repair and contributes to development of endometrial cancer type 1 and development of endometrial cancer in Lynch syndrome. Reduced expression of genes such as *APC*, *CHFR*, *Sprouty 2*, *RASSF1A*, *GPR54*, *CDH1*, and *RSK4* through a similar mechanism of hypermethylation has also been found in endometrial cancer. Suppression of gene expression by miRNAs also occurs in endometrial cancer, and expression of the miRNA itself may be increased or decreased by promoter methylation, based on differences between normal and cancerous endometrial tissue, and may contribute to canceration of the endometrium.

The studies described above have contributed to gradual elucidation of the mechanisms of canceration in the endometrium, but the findings do not seem to be applicable to all cases. Thus, it is unclear whether there is a common underlying mechanism or whether the apparently similar pathological conditions among cases of endometrial cancer are actually somewhat different and new classifications are needed. More knowledge of the canceration mechanism should reveal new therapeutic targets and discovery of new drugs. This is especially true for miRNA targeting since siRNA modulation of miRNA has been established *in vitro* and a similar approach *in vivo* may prevent progression of canceration caused by silencing of miRNA. Drugs that inhibit DNA methyltransferases and thus cause gene demethylation have also been developed, but improved targeting of these drugs is needed for practical clinical use.
